# Are unpopular children more likely to get sick? Longitudinal links between popularity and infectious diseases in early childhood

**DOI:** 10.1371/journal.pone.0222222

**Published:** 2019-09-10

**Authors:** Vidar Sandsaunet Ulset, Nikolai Olavi Czajkowski, Brage Kraft, Pål Kraft, Ellen Wikenius, Thomas Haarklau Kleppestø, Mona Bekkhus

**Affiliations:** 1 Department of Psychology, University of Oslo, Oslo, Norway; 2 Promenta Research Centre, Department of Psychology, University of Oslo, Oslo, Norway; 3 Institute of Clinical Medicine, University of Oslo, Oslo, Norway; Bielefeld University, GERMANY

## Abstract

Social stress and inflammatory processes are strong regulators of one another. Considerable evidence shows that social threats trigger inflammatory responses that increase infection susceptibility in both humans and animals, while infectious disease triggers inflammation that in turn regulates social behaviours. However, no previous study has examined whether young children’s popularity and their rate of infectious disease are associated. We investigated the longitudinal bidirectional links between children’s popularity status as perceived by peers, and parent reports of a variety of infectious diseases that are common in early childhood (i.e. common cold as well as eye, ear, throat, lung and gastric infections). We used data from the ‘Matter of the First Friendship Study’ (MOFF), a longitudinal prospective multi-informant study, following 579 Norwegian pre-schoolers (292 girls, median age at baseline = six years) with annual assessments over a period of three years. Social network analysis was used to estimate each child’s level of popularity. Cross-lagged autoregressive analyses revealed negative dose–response relations between children’s popularity scores and subsequent infection (*b* = –0.18, CI = –0.29, –0.06, and *b* = –0.13, CI = –0.23, –0.03). In conclusion, the results suggest that children who are unpopular in early childhood are at increased risk of contracting infection the following year.

## Introduction

Research suggests that there is a strong social gradient in the risk of contracting infections [1]. Children from families with low socio-economic status are at higher risk of contracting infections from several pathogens, and experience a greater overall burden of infection [2,3]. This phenomenon may in part be explained by differences in parental health behaviours [4], access to healthcare [5] or environmental stress and hazards [6]. However, animal studies and those in adult human populations provide evidence for bidirectional influences between social status and the immune system [7]. This result suggests a co-regulation between social behaviour, inflammatory processes and infection that is deeply rooted in our evolutionary history [8,9]. For example, experimental animal studies demonstrate that a decrease in social rank is followed by subsequent changes in gene expression and immune system activity, that in turn increase infection susceptibility in macaques [9]. Similar results have been found in studies of humans. For example, exposure to social threats and rejection in early childhood have been linked with chronically elevated inflammation [10]. In human adults and in animals, chronic inflammation increases the risk of attracting a cold [11,12]. One might speculate if early social experiences may influence the long-term health of children via parallel biological mechanisms, such that social stress in early childhood increases the risk of attracting infections. To our knowledge, no studies to date have examined whether social status as perceived by peers is associated with young children´s risk of attracting an infectious disease.

The preschool years are a time when children’s immune systems develop defence strategies against their local disease ecology [13]. During this period, most children encounter their first experiences of social inclusion and rejection, possibly setting the stage for the first social influences on infection susceptibility. Typically, young children make their first friends, and form social hierarchies already from preschool age [14–17]. In these social hierarchies, popular children typically engage in more prosocial behaviours such as helping, sharing and comforting other children. However, children who are perceived as being popular by their peers also engage in more physical, verbal and relational aggression [18]. Preschool and the transition to school can be harsh and stressful for young children. Social exclusion is extremely common [19], and both verbal and physical aggression occur frequently, especially at times when social dominance relationships are negotiated [20]. Day-care attendance and the transitioning to school is associated with increased cortisol excretion [21–23], and particularly those children who are perceived as unpopular by their peers exhibit higher cortisol levels and experience more aggressive interactions and peer rejection [24]. In the long term, the stress following frequent subjection to physical, verbal or relational aggression may alter immune functioning, and can ultimately lead to a variety of mental and somatic health problems in both animals and humans [25–27].

Experimental studies provide evidence that subordination to higher-ranking individuals affects immune functioning and increases susceptibility to infection in animals [9]. The primate brain tends to appraise social devaluation, exclusion and rejection as threat information [3]. Such social stressors may cause an up-regulation of inflammatory processes, as the immune system prepares to deal with potential consequences of either fight or flight, such as tissue damage. Exposure to an episode of acute social stress causes an immediate and significant up-regulation of the inflammatory response, that is worth the physiological cost because it increases the likelihood of survival [28]. This is related to the release of epinephrine from the adrenal medulla, which in turn seem to activate the immune system to release cytokines into the bloodstream [29]. However, increased epinephrine levels also cause the hypothalamus to activate the hypothalamic-pituitary-adrenal axis (HPA-axis), one end result being increased release of cortisol from the adrenal cortex. One effect of cortisol is the down-regulation of the acute high levels of cytokines, which is important since cytokines have a cytotoxic effect (e.g. they may kill or damage nerve cells).

However, when stressors (e.g. physical aggression) occur frequently, several negative consequences may occur. The chronically increased levels of cytokines cause long-term activation of the inflammatory response, which represents a state of allostasis and “wear and tear” of the body. Also, immune cells become less sensitive to cortisol, meaning that high levels of cortisol are no longer able to down-regulate the activity of the immune system. That is, immune system cells develop glucocorticoid resistance and continue to produce and release cytokines into the bloodstream [12]. Experimental studies show that mice develop glucocorticoid resistance when they are repeatedly exposed to social stress [30], which in turn is accompanied by less social approach behaviour and higher levels of subordination to aggressive individuals [31]. Humans may also develop glucocorticoid resistance. One study found that healthy adults demonstrated glucocorticoid resistance following a stressful life event, and that those who developed glucocorticoid resistance had higher levels of proinflammatory cytokines, and were at greater risk of catching a cold [12]. Hence cortisol is no longer able to turn the immune system either on or off, but the immune response is instead chronically activated on a heightened but not maximum level (tonic rather than phasic function) [32]. This level is high enough to represent a physiological toll on the cells, but not adequate to fight real threats when they are experienced (e.g. bacteria and viruses). This is why chronic exposure to social stressors may represent an increased risk of attracting infectious diseases.

Conversely, an infectious disease may also influence children’s social behaviour. Historically, infectious disease puts humans and other animals in a very vulnerable situation, where it is important to stay away from harmful individuals, but also to elicit care and support from close others [7]. This notion is supported by findings that adults who are experimentally exposed to endotoxins that trigger inflammation show higher levels of social pain-related neural activity [33] and report increased feelings of social disconnection compared with control participants [34]. Other experimental studies suggest that inflammation may lead to a general sensitivity to both positive and negative social cues [35], and that inflammation increases the perceived need to be with significant others [36]. Currently, no studies have investigated whether infection in childhood is linked with children’s social development; however, a large population-based study has identified associations between a wide range of infectious diseases in childhood and a subsequent increase in the risk of being diagnosed for a mental disorder [37].

This response to infection may be linked with the activity of pro-inflammatory cytokines that activate anhedonia, fatigue, dysphoria and changes in social approach behaviours, also termed sickness behaviours [38,39]. For example, injections that stimulate the release of cytokines have a negative effect on social exploration in mice [40], and when the cytokines interferon-*a* and interleukin-2 is administered to humans (usually as treatment for cancer or hepatitis C) they cause fatigue, sleepiness, irritability, cognitive changes and loss of appetite [41]. Analogous mechanisms may operate in young children. For example, it may be that sickness behaviours affect children’s social functioning in two reinforcing ways. First, feelings of social disconnection, fatigue and dysphoria may lead to avoidance of other children, particularly peers who are not perceived as close friends. Second, some authors have proposed that sickness behaviours may serve as a signal that warns other children of contamination risk, possibly leading classmates to avoid individuals with infectious diseases [42,43]. However, very few empirical studies have investigated whether children actually avoid sick peers. The only empirical evidence to our knowledge is an experimental study finding that 6 and 7 year old children, but not 4 and 5 year olds, avoided proximity to and contact with peers who they were told were sick [43]. It is possible that this dynamic arise because of the evolved ‘behavioural immune system’ that is biased towards the avoidance of pathogens, and hence elicits a strong withdrawal response from possible contamination sources [44–46], to prevent pathogens from entering the body [47,48]. This avoidance, in turn, can contradict children’s evolved propensities for rough-and-tumble-play, social approach and connection [49]. Therefore, there may be a complex evolutionary trade-off between selection for social approach behaviours and for the avoidance of pathogens. Because pathogens such as bacteria, viruses and parasites have shaped the evolution of all organisms, it has been argued that the large individual cost of infection has pushed for a bias towards pathogen avoidance in humans [46,50].

The main goal of this study is to examine the bidirectional longitudinal links between popularity (as an indicator of social status) and the risk of contracting common infectious diseases. On the one hand, infections in early childhood may be associated with social withdrawal and rejection, that in turn may affect children’s social status development. On the other hand, and in parallel with mechanisms observed in the animal and immunological literature, low social status may increase infection risk via immunological and endocrine pathways. There may also be a bidirectional dynamic between infection and social status, in which self-reinforcing cycles between health and social status may cause upwards and downward ‘spirals’. Thus, in a similar vein with findings from animal studies and the few studies on adult populations, we expect to find negative bidirectional associations between popularity as perceived by peers and infectious diseases that are common in early childhood.

## Methods

### Ethics statement

The data collection has received full ethics clearance from the Regional Committees for Medical and Health Research Ethics of Norway (2.2005.1665). The parents of the children who participated in this study provided an informed written consent.

### Participants

**Fig 1:**
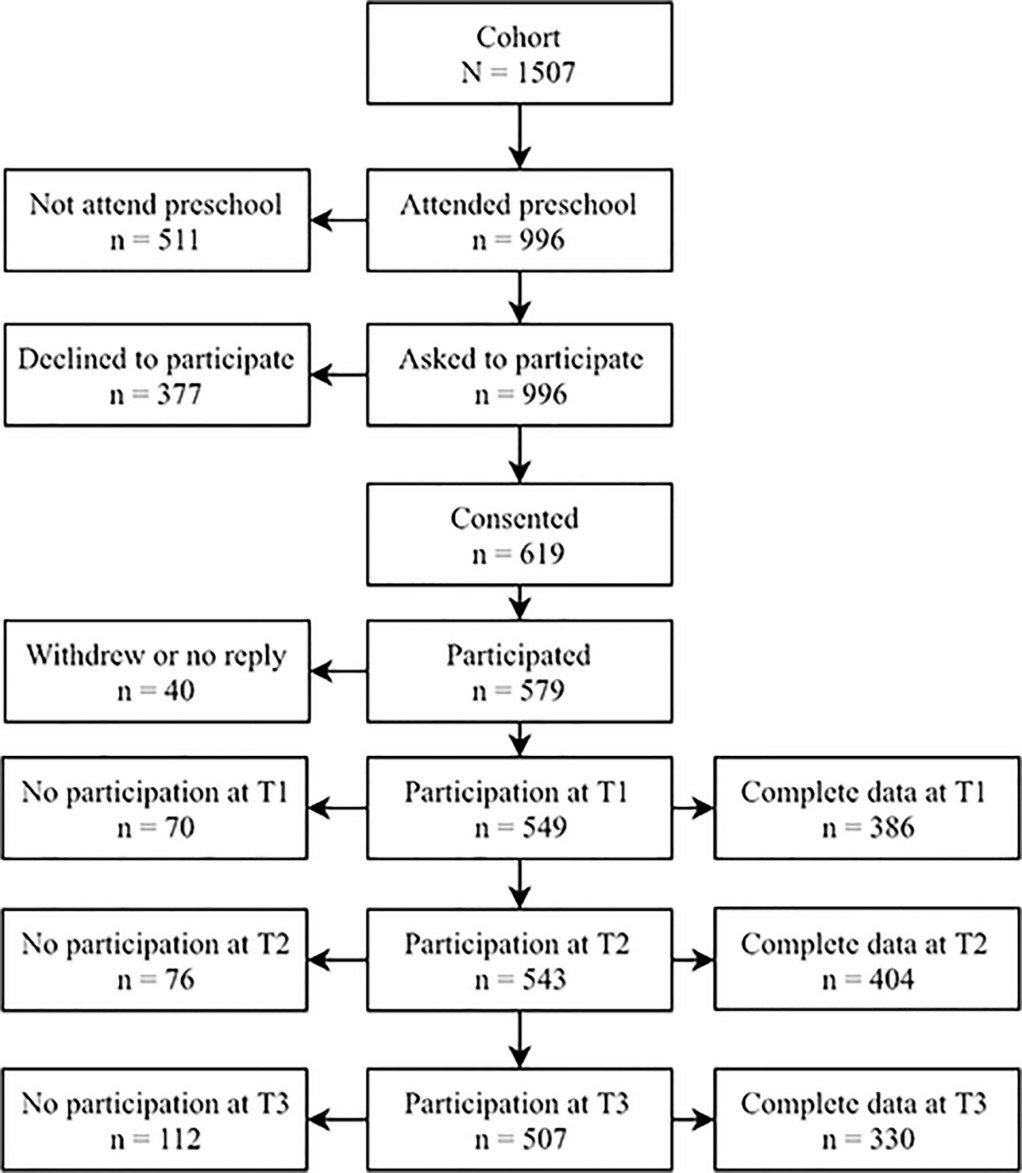
Sample recruitment and follow-up.

The 579 participating children in this multi-informant study were part of the Matter of the First Friendship Study [51,52], a longitudinal study that took place in two suburban Norwegian municipalities with a total population of 24 000. The sampling frame was representative of Norway and comprised children born between 2000 and 2005 that attended one of the 33 day-care centres and 13 primary schools in the two municipalities. A total of 996 children were eligible to participate because they attended one of the day-care centres, while 619 children (62%) were given informed written consent by their parents to participate in the study. As illustrated in Fig 1, a total of 579 participants were registered with at least one response and included in the social network analyses, while 447 participants (243 girls) were registered with complete data on at least one time point and were included in the autoregressive cross-lagged analysis. At the time of the first data collection, the children’s age ranged between 24 and 90 months (mean age = 64.14 months, SD = 15.96).

Parents responded to mailed questionnaires regarding their children’s health. To assess the structures of their social networks and each child’s level of popularity within his or her network, the children were interviewed in their day-care centre or school. In this sample, the maternal education level was higher than the national average, with 45% of mothers having some form of tertiary education. The mean age of the mothers was 35 years (SD 4.7), and 93% were married or cohabitated with a partner. The median income was slightly above the national average for households with children, and slightly higher than the regional average for the municipalities where the families lived.

### Children’s infections

Parents replied to mailed questionnaires, where they provided information about their child’s health. Parents were asked, ‘Has your child experienced one of the six following health problems? If yes, how many times over the previous year?’ (1) eye infection, (2) ear infection (e.g. otitis media), (3) throat infection, (4) lung infection, (5) gastrointestinal tract infection and (6) common colds. To establish a baseline, parents were asked to complete the same list of questions, but retrospectively for the time that their child had just enrolled in day care. A composite score for the mean number of infections for three developmental periods was computed: (1) Baseline, based on parent’s retrospective accounts of their children’s infections at the time they enrolled in preschool, (2) Time 2, based on data collected when children were between 4 and 8 years, and (3) Time 3, based on data collected when the children were 5–9 years. The frequency of infection was coded as 0, 1, 2, 3 or ‘4 or more’, and the items correspond to those used in previous studies of infections in preschool [53–55]. At each assessment, parents were asked whether the child had any serious or long-term illnesses. The presence of a serious or long-term illness was coded 1 for “yes” and 0 for “no”.

### Children’s popularity

**Fig 2:**
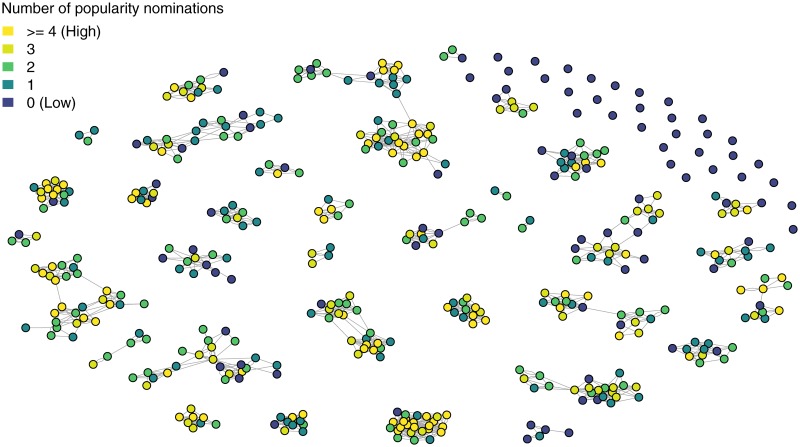
Social networks at Time 1 (N = 447). Note. Nodes represent children. Colors indicate popularity score which is the number of receiving popularity nominations. For parsimony, edges indicate an undirected social tie between two children. Only children with complete data are represented.

Popularity was assessed using a well-known and validated peer-nomination technique [56]. Trained research assistants interviewed the children in their preschool or school, in a separate and quiet room. For children aged two through six years (still in preschool), we used photographs of the children in their group and a cardboard bus for illustration, as the children were given the following instructions: ‘We will now pretend-play that we are going for a trip in a bus. You can sit behind the bus driver. Who do you want to bring on the bus trip?’ The children were asked to place their own picture behind the driver, and then to nominate up to five classmates to bring on the bus. The children indicated who they wanted to bring along by placing the nominated children’s pictures on the cardboard bus. After transitioning to elementary school, children (age seven through nine years) were asked to nominate whom they would like to bring on a bus trip by saying their names. They were not restricted to nominating their classmates, and some children had friends in other classes or from their old day-care group. We operationalized popularity as the total number of nominations received [57], standardized by the number of total possible nominations (i.e. the size of their social network). Fig 2 illustrates the distribution of popularity scores across social networks.

### Analytical strategy

All statistical models were fitted using the software package Mplus [58]. To examine the bidirectional links between popularity and infectious diseases, we used autoregressive cross-lagged analysis. This approach is in accordance with previous studies that used such analysis with popularity network data [59]. The frequency of infectious diseases and popularity at Time 3 were regressed on assessments of the same variables at Time 2, while Time 2 measures were regressed on Time 1 measures. We allowed for residuals of infection and popularity at the same time points to be correlated. To correct for a skewed distribution of infection scores, we employed a robust maximum likelihood estimator. There were no missing data in the popularity scores. Missing data in the infection variables (16.77%) were handled using a full information maximum likelihood procedure.

Popularity scores were standardized by social network size (i.e. the number of possible nominations), before they were entered into the autoregressive cross-lagged model. We used community detection [60] to identify the sizes of the social networks. Social network analyses were carried out in R studio using the igraph package [61].

## Results

The distributional properties and descriptive statistics of the study variables are presented in Table 1. There was a decrease in infection scores between Times 1 and 3 (40.30%). For popularity scores, there was a small increase (6.59%) between Times 1 and 3. Popularity scores were evenly distributed across 0, 1, 2, 3 and 4 or more nominations. On average, 17.60% of the children received zero popularity nominations, 23.53% received one nomination, 22.00% received two nominations, 15.50% received three nominations and 16.80% of the children received four popularity nominations or more (see Fig 2). Children´s age was negatively correlated with infection at T2 (b = -0.19, p < 0.00), and positively correlated with popularity at T1 (b = 0.21, p < 0.001) and T2 (b = 0.10, p = 0.03). Independent samples t-tests revealed no significant differences between girls and boys in popularity scores. However, boys showed higher scores on infection at T2 (boys M = 0.57, girls M = 0.49, F = 6.55, p = 0.02), and at T3 (boys M = 0.37, girls M = 0.43, F = 2.00, p = 0.40). Boys also showed higher scores on infection at T1 (boys M = 0.72, girl M = 0.62) but this difference was not significant (p = 0.06).

**Table 1:** Pearson´s bivariate correlations and descriptive statistics.

	1	2	3	4	5	6
1. Infections T1						
2. Infections T2	0.29***					
3. Infections T3	0.03	0.23***				
4. Popularity T1	-0.06	-0.18***	-0.06			
5. Popularity T2	-0.03	-0.08	-0.15**	0.34***		
6. Popularity T3	0.07	-0.04	-0.07	0.27***	0.27***	
N	386	404	330	579	579	579
Mean	0.67	0.53	0.40	1.67	1.75	1.78
SD	0.54	0.34	0.29	1.39	1.91	1.37
Min–Max	0–4	0–2.5	0–2	0–4	0–4	0–4

*p < 0.05,

**p < 0.01,

***p < 0.001

**Fig 3:**
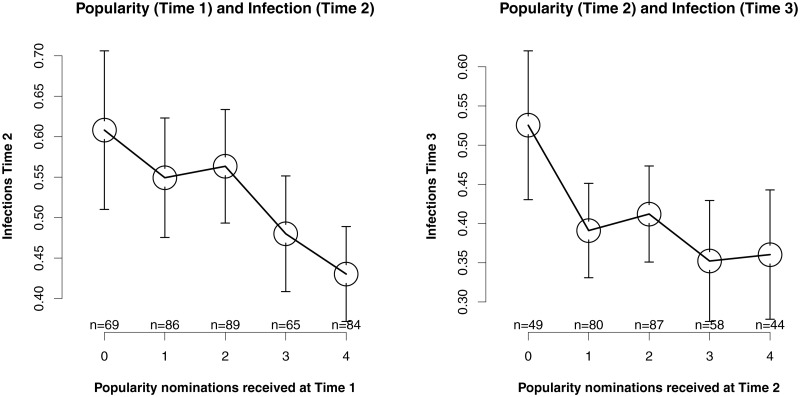
Dose-response relations between popularity and infections at Time + 1. Note. Error bars in the plot indicate a 95% confidence interval.

Table 1 shows the bivariate correlation coefficients between all variables. On the bivariate level, there were small to moderate positive correlations between infection at Times 1 and 2, and between infection at Times 2 and 3. All time points of popularity were moderately and positively correlated with each other. There was a small negative association between popularity at Time 1 and infection at Time 2, and between popularity at Time 2 and infection at Time 3. The prospective correlations between popularity and infections at the following year, are illustrated with line plots in Fig 3. The line plots in Fig 3 reveal a dose–response relation between popularity and infections. The infection scores of children who received zero popularity nominations (*M* = 0.57), were on average across time points 28.33% higher than for those children who received four or more popularity nominations (*M* = 0.39).

**Fig 4:**
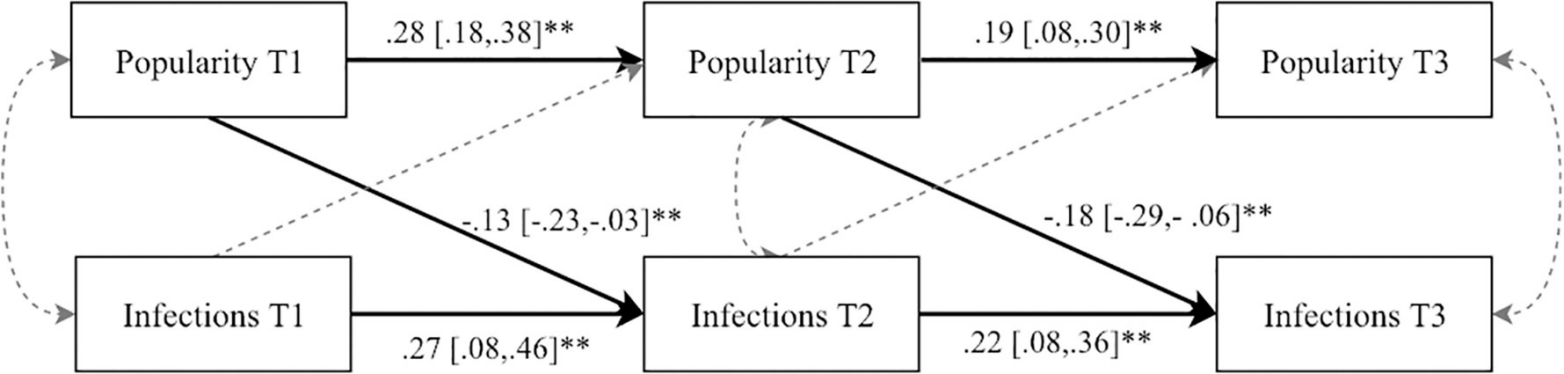
Autoregressive and cross-lagged relations between children´s popularity and infectious diseases. Note. For simplicity, only significant standardized regression coefficients are shown, with confidence intervals in brackets. Significant paths are highlighted with solid arrows.

*Autoregressive cross-lagged analysis*. The results from the autoregressive cross-lagged model examining bidirectional relations between popularity and infections are shown in Fig 4. The model fit was excellent (χ^2^ = 5.60, df = 14, *P* = 0.23, CFI = 0.98, RMSEA = 0.033, Standardized Root Mean Square Residual = 0.023). At both Times 1 and 2, high popularity scores predicted lower levels of infections with respect to the following measurement. One standard deviation increase in popularity was associated with a 0.13 (Time 2) and 0.18 (Time 3) standard deviation decrease in infection. There were no residual associations between infections and popularity within the same time points, nor any significant cross-lagged effects of infections on popularity on the subsequent measurement wave. Additional analyses showed that controlling for children´s age and gender, or whether the children had a serious or long-term illness, did not impact the estimates of the model (changes in the standardized coefficients ranged from *b* = 0.01 to 0.03). At Time 1, parents of 23 children (2%) reported that their child had a serious or long-term illness.

## Discussion

The main aim of this study was to examine bidirectional associations between children’s popularity as perceived by their peers, and the frequency of a variety of common infections as reported by their parents. Results from autoregressive cross-lagged analyses suggest that high popularity scores were associated with low levels of infection in the following assessment. Surprisingly, we found no concurrent links between popularity and infection within the same time points, nor any prospective effects of infections on popularity scores. There were dose–response associations between popularity and subsequent infection, a result which is consistent with previous studies demonstrating a graded link between social status and a wide range of health outcomes, including infection [1,6,62].

Our finding that children’s popularity is associated with subsequent infection risk is in accordance with extensive evidence from animal studies showing that reductions in social status changes inflammatory processes and increases infection susceptibility in animals [3]. Previous studies found that social stress in childhood is related to subsequent inflammatory changes [10], and we add to this knowledge by demonstrating links between popularity and rates of contracted infectious diseases.

Moreover, we found links between popularity and infectious diseases the following year, but not the concurrent year. These long-term links may reflect a delayed response of infection susceptibility to social stress, similar to those observed in animal models [63]. However, another explanation might be that the results are influenced by developmental timing effects. Some researchers have suggested that infections and social experiences have more implications for later development if they occur early in life [13]. It is possible that the interwoven processes between social and immunological development are in place very early on in life, and that there are different developmental windows with somewhat unique challenges to both health and social systems. As such, the strength and relative directionality of their interrelations may vary as well over time. This would be an important point to examine in future studies.

Our finding that popularity as perceived by peers is associated with infectious diseases in childhood suggests that early social dynamics may precipitate long-term differences in health. Because our study was observational and did not involve measures of cortisol or immune functioning, we can only speculate on the causal mechanisms that underlie our findings; however, we outline some possible mechanisms here.

From an evolutionary perspective, children who are excluded from their group have historically been under major risk of being attacked and wounded by predators. To anticipate tissue injury, the immune system therefore reacts to social exclusion by up-regulating pro-inflammatory gene expression, and as a consequence down-regulating anti-viral responses. This process is a highly conserved transcriptional response to social adversity in non-human primates [64] and adult humans [65]. This conserved transcriptional response to social adversity may have become maladaptive in modern educational settings because prolonged pro-inflammatory responses to social exclusion may, in the long run, promote health problems [42,66].

For some children, educational settings set the stage for experiences of social stress, bullying, subordination and rejection [67]. At the same time, children in preschool and school are confined to crowded settings where they are exposed to commutable pathogens and are at increased risk of contracting infections [53]. The risk of being attacked and wounded by predators or a fellow human is low compared with the historical contexts where humans evolved. A conserved transcriptional response to social adversity may be maladaptive in modern society, and only contribute to exacerbating negative effects of low social status (such as lack of social support), by also increasing the risk of contracting an infection. This supposition, however, remains speculative, and needs to be investigated with both prospective and experimental studies, incorporating measures of gene expression.

The experience of being unpopular may increase allostatic load when children repeatedly experience social rejection or aggression from their peers. In non-human primates, dominance–subordination relationships are frequently maintained by the dominant individuals’ aggression, threats and intimidation. This pattern can also be observed in children because children who engage in higher levels of physical, verbal and relational aggression often are perceived as being popular by their peers [18]. When people experience social threats, they respond with highly adaptive sympathetic responses leading to activation of their fight or flight response [68]. When these responses are frequently repeated, stress allostasis becomes chronically elevated, ultimately down-prioritizing anti-inflammatory and -viral responses as energy expenditure is reallocated to deal with immediate threats [13]. Such exposure to social threats in early childhood may lead to epigenetic changes, inflammatory changes and dysregulation of the hypothalamic–pituitary–adrenal (HPA) axis [27]. Future studies should examine whether associations between popularity and infection are mediated by indicators of stress, such as the HPA hormone cortisol.

To our surprise, we did not find significant links between infection and subsequent or concurrent popularity. According to the behavioural immune system hypothesis, children would distance themselves from sick individuals to prevent pathogens from entering their bodies [44], while infected children would withdraw from social situations because of cytokine-induced sickness behaviours [39]. One possible explanation for our non-finding may be that avoidance of sick peers is mainly temporary, and that most children who have been sick are included again as soon as the infection has passed. In addition, sick children will normally be kept at home by their parents, limiting the exposure to peers during the course of infection.

A major strength of our study was the use of a multidisciplinary approach, including parental questionnaires and extensive interviews with the children. Participants were followed prospectively throughout preschool and elementary school, and our sample represents a variety of forms, sizes and models of preschools, to date the largest prospective study with child interviews. However, some limitations should be mentioned. Parents did not report information about the duration or severity of the infections. We did not measure the possible causal mechanisms that underlie our findings, in terms of biological markers of inflammation, stress or genetic mediators. Our study is a prospective longitudinal one, meaning that we cannot rule out unmeasured confounders, such as systematic variation in genetic susceptibility to both infections and popularity. In particular, it is possible that children who are highly popular also have high degrees of fitness-related traits that make them popular, such as intelligence and emotional stability, and that these traits are, in turn, genetically related to other fitness-related characteristics such as immunological functioning. Hence, correlations between popularity and effective immune functioning can, at least in part, have shared genetic influences (See [69]). Importantly, however, our finding is a strong indicator of a dose–response relation between popularity and infection, demonstrates the direction of this relationship over time, and converges with experimental animal studies that demonstrate strong links between social status and infection susceptibility [70].

## Conclusion

Our findings suggest that popular children are less likely to contract infectious diseases. We did not find any associations between infectious disease and subsequent popularity, nor did we determine any concurrent associations between popularity and infectious disease. Attempts to understand the early development and maintenance of social differences in health should consider children’s social status as perceived by their peers. Future studies should examine whether biological regulation mediates this link between children’s popularity and subsequent risk of infection.
